# Perspectives on early insights: pediatric cancer caregiving amidst natural calamities – A call for future preparedness

**DOI:** 10.3389/fpubh.2023.1319850

**Published:** 2024-01-09

**Authors:** Damiano Rizzi, Giulia Ciuffo, Firdaous El Gour, Jinane Erradi, Lavinia Barone, Chiara Ionio

**Affiliations:** ^1^Fondazione Soleterre Strategie di Pace ONLUS, Milan, Lombardy, Italy; ^2^Psychology of Trauma Research Unit, Department of Psychology, Catholic University of the Sacred Heart, Milan, Lombardy, Italy; ^3^Department of Brain and Behavioral Sciences, University of Pavia, Pavia, Lombardy, Italy; ^4^Fondazione Policlinico San Matteo IRCCS, Pavia, Italy; ^5^Centre Hospitalier Universitaire Mohammed VI, Marrakesh, Morocco; ^6^Department of Psychology, Catholic University of the Sacred Heart, Milan, Italy

**Keywords:** natural disaster, earthquake, Morocco, mental health, cancer, healthcare

## Abstract

Natural disasters cause immense damage and disruption to the environment, human lives, and property, posing a threat to safety and well-being. These disasters annually affect individuals and communities, severely impacting mental health. Research indicates a significant link between catastrophic events and an increased risk of mental disorders, including anxiety, depression, substance use, and post-traumatic stress disorder (PTSD). Individuals with chronic conditions, like cancer patients, are particularly vulnerable post-disaster due to disrupted healthcare services. The recent earthquake in Morocco highlighted the urgent need for continued care, especially for vulnerable populations living in poverty. Soleterre Foundation’s interventions focus on supporting young cancer patients and their families, emphasizing psychological support following the earthquake. Effective disaster response needs coordinated efforts, clear roles, communication, and standardized healthcare procedures, especially for vulnerable groups like cancer patients. Education programs for patients and clinicians are vital for disaster preparedness. Communication challenges and lack of medical history further emphasize the need for well-defined disaster preparedness plans and continued care guidelines for cancer patients.

## Introduction

A natural disaster is a catastrophic event that occurs due to natural processes of the Earth, resulting in significant damage, destruction, and disruption to the environment, human life, and property. These events are often sudden, intense, and uncontrollable, causing widespread devastation and posing a threat to the safety, well-being, and livelihoods of affected populations. Disaster is defined by the World Health Organization (WHO) as a sudden ecological catastrophe or a phenomenon that necessitates outside help (1). Every year, disasters strike, adversely affecting individuals and communities, ultimately undermining their mental health and well-being (2). A recent systematic review and meta-analysis (3) suggested that experiencing a catastrophic disaster is connected to an elevated risk of mental disorders in the general population. The main mental health disorders associated with the disaster catastrophe in most of the studies included were generalized anxiety disorder (GAD), depression, substance use disorder, adjustment disorder, and post-traumatic stress disorder (PTSD). The prevalence rates for these mental health disorders ranged from 5.8 to 87.6%. Moreover, the likelihood of experiencing psychological morbidity heightened due to the relocation and disruption of essential services. During the first year following a disaster, psychological morbidity commonly afflicts approximately 30 to 40% of the population, and there’s a probability of a lingering disease burden becoming chronic (4, 5). However, there’s a scarcity of research concerning the influence of disasters on psychopathology (5).

Within the context of natural disasters, the health system is crucial in saving lives and providing social support to vulnerable individuals (6). The initial responses after a disaster emphasize the provision and distribution of critical needs like food, shelter, water, and meeting other essential requirements for the affected population. This includes managing injuries resulting from the disaster, as well as responding to and treating infectious diseases and acute conditions (7). Among those affected by these events, individuals with chronic conditions, such as patients with cancer, stand out as a particularly vulnerable group, facing a range of issues post-disaster (8, 9). Indeed, in the wake of medical facilities collapsing and the overwhelming burden on remaining hospitals and healthcare centers, ensuring continued care for chronic patients became a paramount issue (10). Indeed, insufficiently managed chronic conditions can pose a risk to the community’s life and overall well-being immediately following such disasters. However, historically, their treatment has not been acknowledged as a priority in public health or medicine (11). Individuals battling chronic conditions, such as cancer face numerous obstacles and diverse requirements during and following disasters. It’s essential for cancer care to persist throughout these challenging times. Cancer represents an ongoing global challenge (12). According to a recent analysis conducted by the Global Burden of Disease, there were an estimated 17.2 million new cancer cases and 10 million cancer deaths globally, in 2019 (13). While the rates of cancer occurrence vary from one country to another, it represents a shared burden across all societies (14, 15). For this reasons, the health system should prioritize bolstering hospital surge capacity and ensuring the availability of human, financial, and medical equipment resources to effectively address catastrophic conditions (16).

### Morocco earthquake: what impact on the health system?

The powerful earthquake that hit Morocco’s High Atlas Mountains the 8 September 2023 had a magnitude of 6.8, which is the biggest to hit the North African country in 120 years. As reported by the news (17), the earthquake resulted in a tragic loss of life, claiming the lives of at least 2.946 individuals, primarily in Marrakech and five provinces near the earthquake’s epicenter, as reported by Morocco’s interior ministry. Furthermore, a minimum of 5.674 individuals suffered injuries, with 1.404 in critical condition, according to officials. The final count of casualties is likely to rise considerably, given the challenges faced by rescue teams in reaching remote mountain villages that bore the brunt of the devastation due to obstructed roads strewn with boulders. The World Health Organization (WHO) conveyed that the earthquake has impacted over 300.000 individuals across the nation.

The healthcare access for the Moroccan population has significantly expanded in under 15 years, although there remains a substantial journey ahead to attain Universal Health Coverage (UHC) (18). The Ministry of Health and Social Protection in Morocco (MSPS: Ministère de la Santé et de la Protection Sociale) has implemented a diverse range of strategies and interventions. These efforts primarily revolve around addressing the Social Determinants of Health (DSS) to enhance the overall health of the Moroccan populace and diminish regional disparities and health inequalities. However, much effort is still needed to reduce such inequities (19). Indeed, a quantitative survey on access to care carried out between 2010 and 2011 highlighted how over 80% of the participants found it very challenging to access healthcare facilities. Moreover, the quality of care was rated as substandard or insufficient (20).

Much of the population affected by the earthquake lives in deep poverty. With hospitals severely overstretched because of the earthquake, it is difficult to predict how continuity of care, including psychological care, will be ensured for the chronically ill.

### Soleterre foundation intervention in Morocco

Soleterre Foundation is a non-profit foundation and non-governmental organization (NGO) that has been operating in Morocco since 2002, where it runs an international paediatric oncology programme in collaboration with the Mohammed VI Hospital, in Marrakech, which represents the hub for the whole south of Morocco. Soleterre’s community home shelter (*Dar Amal House*), opened in 2022 in Marrakech, near Mohammed VI Hospital, accommodates young cancer patients and their families who otherwise often must travel up to 1,500 km to reach the hospital and undergo chemotherapy treatment. The house commonly hosts 30 patients and their families. After the earthquake, many children from the affected neighboring villages were hosted by Soleterre and the home currently accommodates 49 children (aged between 2 and 15 years) with an oncological disease. Soleterre provides psychological support to these children and their families, as well as managing these logistical aspects. Currently, in the aftermath of the earthquake, the Foundation’s goal is to train local psychologists to provide psychological support in the hospitals and villages affected, addressing the country’s shortage of trained psycho-oncologists. Indeed, the impact of possible multiple traumas, cancer and the earthquake, on the mental health of these children should not be underestimated at this particular time.

### Getting started

In the few days after the earthquake, Soleterre Foundation started to collect some first raw data about the caregivers’ conditions and well-being. Data collection was conducted in an emergency situation, so the sample size was not predetermined. Participants were given the opportunity to decline participation in the study, which was consistent with ethical standards of research practice. They were repeatedly assured that participation in the study would not affect the services they received. Respondents were first asked to provide sociodemographic information, questions related to the earthquake experience (such as location and its impact), the number of days caregivers spent at the hospital, and their knowledge about future accommodations—particularly relevant given the loss of homes for most participants. The assessment of caregivers’ mental health status was conducted encompassing various domains (i.e., anxiety, depression, coping mechanisms). These inquiries were presented in an interview format, allowing caregivers to express their feelings openly and share their experiences of suffering. Soleterre psychologists crafted the questionnaire by drawing on their observations within the pediatric service and considering how the earthquake impacted the individuals involved. In developing the questions, they closely examined the unique situations and challenges faced by people affected by the earthquake within the pediatric service. The aim was to tailor the questionnaire to capture the specific experiences and emotions of caregivers.20 caregivers were interviewed, the sample size was determined by the capacity of the service, which accommodated 20 families. Their main socio-demographic characteristics are summarized in Table 1.

**Table 1:** Caregivers’ socio-demographic characteristics.

Socio-demographic characteristics	Value
Age (mean years ± SD)	49.29 ± 14.70
Nationality (%)	
Moroccan	100
Region (%)	
Marrakech Safi Al Haouz	100
Marital Status (%)	
Married	85
Widow	15
Number of children (mean years ± SD)	3.60 ± 1.231
Days spent at the hospital (mean years ± SD)	5.79 ± 2.463
Relatives left behind at the village (%)	
Yes	65
No	35

Table 2 summarises the caregivers’ responses regarding their mental health status.

**Table 2:** Cargivers’ mental health status.

Mental health status	Value
Anger (%)	
Low	30
Moderate	25
High	45
Fear (%)	
Low	15
Moderate	50
High	35
Restlessness (%)	
Low	10
Moderate	50
High	40
Anxiety (%)	
Low	15
Moderate	50
High	35
Sadness (%)	
Low	/
Moderate	30
High	70
Sleep quality (%)	
Sleep well	35
Suffer from insomnia	65

Caregivers showed a deep concern for the health of their loved ones. However, their enduring faith in religion and spirituality played a crucial role in strengthening their psychological resilience in the midst of the disaster.

## Conclusion

These initial data suggest the need for early and targeted intervention to prevent long-term psychopathological outcomes, particularly given the impact that caregivers’ mental health can have on the young oncological patients. From these raw preliminary data, the quality of sleep seems to play a significant role in how individuals cope with and recover from traumatic situations. Traumatic events can cause disruptions in sleep patterns and overall sleep quality, which can, in turn, affect both physical and mental health. Indeed, addressing sleep-related issues and promoting good sleep hygiene are vital components of trauma recovery and mental health treatment. Therapeutic approaches that focus on improving sleep quality can contribute significantly to the overall well-being and resilience of individuals who have experienced traumatic events. It is important to note that trauma represents a complex situation that needs all-encompassing care. While improving sleep quality is crucial, trauma healing involves many other factors as well. It’s also necessary to address other symptoms like depression, anxiety, and post-traumatic stress disorder (PTSD) with the right therapies and support systems. Indeed, a recent systematic review and meta-analysis (3) suggests that the most common long-term consequences of a disaster are anxiety, GAD and PTSD. Over time, people are affected by disasters in different ways; some people report increased symptoms of anxiety and PTSD (21). Others argue that exposure to a single event or a series of them raised the likelihood of psychological morbidity in similar manners (22). The majority of the studies showed that anxiety, depression, and PTSD were more common psychological diseases associated with temporary housing and evacuation. In addition to relocation, a rise in mental illness was noted as a result of the disruption of essential services, employment, or education (23–26). To successfully address the effects of trauma on mental health, caregivers and young patients with cancer must get holistic treatment that incorporates a variety of therapy modalities. This may involve creating specialized support groups for both patients and caregivers, implementing counseling services tailored to the unique needs of this population, and developing clear protocols for local healthcare professionals to identify and address psychosocial challenges promptly. Moreover, to establish a comprehensive “protective shield” that effectively lessens the impact of socio-ecological shocks, it is crucial to assess both the protective capabilities and shortcomings within the systems. Indeed, the presence of family members can transform adversity into a source of strength, as they contribute to the creation of a meaningful universe. Consequently, the family plays a vital role in serving as an anchor for identification and emotional stability (27). While researchers have traditionally recognized the detrimental effects of acute stress, recent studies have adopted a resilience approach (28, 29). These investigations underscore that traumatic experiences may result in dysfunction, but families can endure, recover, and even prosper in the aftermath of adverse events (30). As emerged by our preliminary data, religion and faith can serve as powerful coping strategies in various ways, providing individuals with a sense of meaning, comfort, and support during challenging times. In applying this knowledge, mental health professionals should approach discussions of religion and faith with sensitivity, respect, and cultural competence. Tailoring interventions to align with an individual’s belief system and incorporating religious or spiritual practices into therapeutic approaches can enhance the effectiveness of coping strategies rooted in faith. Additionally, fostering an open dialog about the role of religion in coping can contribute to a more holistic and person-centered approach to mental health care.

Moreover, while our preliminary findings emphasize the importance of addressing sleep-related issues in the aftermath of the earthquake, it is crucial to underscore additional critical considerations highlighted during our early observations. Indeed, in the wake of the disaster, Soleterre caught the urgent need to strengthen local mental health resources, particularly focusing on the scarcity of trained psycho-oncologists in the country. The earthquake exposed a significant gap in mental health resources, with the shortage of trained psycho-oncologists emerging as a pressing issue. Soleterre Foundation’s commitment to training local psychologists signifies a crucial step toward building sustainable and resilient mental health infrastructure. This intervention not only responds to the immediate needs of caregivers and young oncological patients post-disaster but also lays the groundwork for enhanced preparedness in the face of future calamities. Building local capacity in psycho-oncology is essential for providing ongoing and specialized mental health support to cancer patients and their caregivers, particularly in this situation. The scarcity of trained psycho-oncologists in Morocco is a systemic challenge that requires a multifaceted approach. Soleterre Foundation’s intervention could serve as a model for organizations and policymakers, highlighting the importance of investing in local training programs and initiatives. Collaborative efforts between NGOs, governmental bodies, and educational institutions can contribute to the development of a sustainable framework for mental health care, not only during emergencies but also in routine healthcare settings. In light of the resource limitations in this region of Morocco, it’s imperative to consider practical and realistic solutions for optimizing care for oncology and pediatric oncology patients. While the call for more psycho-oncologists is a valid concern, it may not be the sole solution given the constraints. Leveraging existing resources, partner with local universities and educational institutions to develop specialized training programs in psycho-oncology could enhance the skills of current professionals and create a sustainable pipeline of expertise. Using telemedicine, the feasibility of virtual consultations for psycho-oncology support could be explored. This can extend the reach of mental health services to remote areas, overcoming geographical barriers. It could also be possible to develop online training programs for healthcare professionals and caregivers, making resources accessible beyond physical constraints. Moreover, launch public awareness campaigns to educate the community about the psychological needs of oncology patients and the available support services may reduce stigma, encourage early intervention, and foster a supportive environment.

To guarantee an efficient response, it is essential to synchronize disaster preparedness efforts with all involved parties, establish clearly defined roles, and outline procedures. Clinicians need thorough education regarding disaster response and appropriate care for cancer patients amidst and following disasters. Additionally, it is imperative to create guidelines for maintaining regular care for cancer patients in adverse situations. Following disasters, communication becomes severely constrained, affecting interactions among healthcare providers, between providers and their patients, and among provider agencies and governmental bodies (31). To ensure effective treatment for cancer patients, it is crucial to have reliable communication channels and standardized health protocols (32). Another significant hurdle is the absence of medical records for cancer patients and limited information on cancer treatment in catastrophic scenarios, posing challenges for patient-caregiver interactions. To address this and enhance preparedness, there is a critical need to formulate an action plan that outlines essential care for cancer patients, aligning with established guidelines. Furthermore, educational programs on disaster preparedness should be conducted for both patients and clinicians.

## Data availability statement

The raw data supporting the conclusions of this article will be made available by the authors, without undue reservation.

## Author contributions

DR: Conceptualization, Investigation, Methodology, Project administration, Supervision, Writing – review & editing. GC: Conceptualization, Data curation, Formal analysis, Methodology, Visualization, Writing – original draft, Writing – review & editing. FE: Investigation, Writing – review & editing. JE: Investigation, Writing – review & editing. LB: Writing – review & editing. CI: Conceptualization, Data curation, Methodology, Resources, Supervision, Visualization, Writing – original draft, Writing – review & editing.

## References

[1] WHO/EHA. Disasters & Emergencies Definitions Training Package. Addis Ababa: Panafrican Emergency Training Centre. (2022).

[2] Kreimer A. Social and economic impacts of natural disasters. Int Geol Rev. (2010) 43:401–5. doi: 10.1080/00206810109465021

[3] Keya TA Leela A Habib N Rashid M Bakthavatchalam P. Mental health disorders due to disaster exposure: a systematic review and meta-analysis. Cureus. (2023) 15:e37031. doi: 10.7759/cureus.37031, PMID: 37143625 PMC10153020

[4] WHO. World mental health report: transforming mental health for all. Geneva: World Health Organization. (2022). Available at: https://www.who.int/publications/i/item/9789240049338

[5] Milojevic A Armstrong B Wilkinson P. Mental health impacts of flooding: a controlled interrupted time series analysis of prescribing data in England. J Epidemiol Community Health. (2017) 71:970–3. doi: 10.1136/jech-2017-208899, PMID: 28860201 PMC5754859

[6] Morton PG Fontaine DK Hudak CM Gallo BM. Critical care nursing: a holistic approach. 8th Edition, Philadelphia, USA: Lippincott Williams and Wilkins (2005).

[7] Ozaki A Leppold C Tsubokura M Tanimoto T Saji S Kato S. Social isolation and cancer management after the 2011 triple disaster in Fukushima, Japan: a case report of breast cancer with patient and provider delay. Medicine. (2016) 95:e4027. doi: 10.1097/MD.0000000000004027, PMID: 27368025 PMC4937939

[8] Kessler RC, Group HKCA. Hurricane Katrina’s impact on the care of survivors with chronic medical conditions. J Gen Intern Med. (2007) 22:1225–30. doi: 10.1007/s11606-007-0294-1 17657545 PMC2219784

[9] Heidari M Ghodusi M. The relationship between body esteem and hope and mental health in breast cancer patients after mastectomy. Indian J Palliat Care. (2015) 21:198–202. doi: 10.4103/0973-1075.156500, PMID: 26009674 PMC4441182

[10] Mokdad AH Mensah GA Posner SF Reed E Simoes EJ Engelgau MM. When chronic conditions become acute: prevention and control of chronic diseases and adverse health outcomes during natural disasters. Prev Chronic Dis. (2005) 2:1–4. PMC1459465 16263037

[11] Miller AC Arquilla B. Chronic diseases and natural hazards: impact of disasters on diabetic, renal, and cardiac patients. Prehosp Disaster Med. (2008) 23:185–94. doi: 10.1017/S1049023X00005835, PMID: 18557300

[12] American Association for Cancer Research (AACR). (2023). Cancer progress report. Available at: https://cancerprogressreport.aacr.org/progress/cpr23-contents/cpr23-cancer-in-2023/ (Accessed September 22, 2023).

[13] Global Burden of Disease 2019 Cancer Collaboration Kocarnik JM Compton K Dean FE Fu W Gaw BL. (2022). Cancer Incidence, Mortality, Years of Life Lost, Years Lived With Disability, and Disability-Adjusted Life Years for 9 Cancer Groups From 2010 to 2019: A Systematic Analysis for the Global Burden of Disease Study 2019. JAMA oncology, 8, 420–444. doi: 10.1001/jamaoncol.2021.6987 34967848 PMC8719276

[14] Shahbazi S Heidari M Ghafourifard M. Comparison of direct and indirect methods of teaching breast self-examination–influence on knowledge and attitudes of Iranian nursing and midwifery personnel. Asian Pac J Cancer Prev. (2017) 18:1157–62. doi: 10.22034/APJCP.2017.18.4.1157 PMID: 28548468 PMC5494231

[15] Mahoozi S Heidari M Shahbazi S Naseh L. Influence of training about carcinogenic effects of hookah smoking on the awareness, attitude, and performance of women. Asian Pac J Cancer Prev. (2017) 18:1967–71. doi: 10.22034/APJCP.2017.18.7.1967 PMID: 28749630 PMC5648406

[16] Gorji HA Jafari H Heidari M Seifi B. Cancer patients during and after natural and man-made disasters: a systematic review. Asian Pacific J Cancer Prevent. (2018) 19:2695–700. doi: 10.22034/APJCP.2018.19.10.2695, PMID: 30360593 PMC6291047

[17] Agence France-Presse Reuters and Associated Press. Morocco earthquake: at least 2,000 dead and thousands more injured. (2023). Available at: https://www.theguardian.com/world/2023/sep/09/morocco-earthquakeleaves-panicked-people-sleeping-in-street-reports

[18] National Initiative For Human Development. (2019). Presentation of phase III 2019–2023, platform for the implementation of the third phase of the INDH, from 2019 to 2023. Available at: http://www.indh.ma/wp-content/uploads/2019/09/Livret_INDH_VF.pdf

[19] Zahidi K Moustatraf A Zahidi A Naji S Obtel M. Universal health coverage in Morocco: the way to reduce inequalities: a cross-sectional study. Open Public Health J. (2022):15. doi: 10.2174/18749445-v15-e221222-2022-160

[20] Gruénais ME Amine M De Brouwere V. Qualitative survey on access to care among the population In: National Observatory of Human Development (Ed). Disparities in access to healthcare in Morocco. Rabat: National Observatory of Human Development (2011). 246.

[21] Mason V Andrews H Upton D. The psychological impact of exposure to floods. Psychol Health Med. (2010) 15:61–73. doi: 10.1080/13548500903483478 20391225

[22] Lamond JE Joseph RD Proverbs DG. An exploration of factors affecting the long term psychological impact and deterioration of mental health in flooded households. Environ Res. (2015) 140:325–34. doi: 10.1016/j.envres.2015.04.008, PMID: 25909883

[23] Honda Y Fujiwara T Yagi J Homma H Mashiko H Nagao K. Long-term impact of parental post-traumatic stress disorder symptoms on mental health of their offspring after the great East Japan earthquake. Front Psych. (2019) 10:496. doi: 10.3389/FPSYT.2019.00496/BIBTEX, PMID: 31404309 PMC6675868

[24] Kino S Aida J Kondo K Kawachi I. Persistent mental health impacts of disaster. Five-year follow-up after the 2011 great East Japan earthquake and tsunami: Iwanuma study. J Psychiatr Res. (2020) 136:452–9. doi: 10.1016/j.jpsychires.2020.08.016, PMID: 32948310 PMC7443179

[25] Schwind JS Formby CB Santangelo SL Norman SA Brown R Hoffman Frances R. Earthquake exposures and mental health outcomes in children and adolescents from Phulpingdanda village, Nepal: a cross-sectional study. Child Adolesc Psychiatry Ment Health. (2018) 12:54. doi: 10.1186/s13034-018-0257-9 30598695 PMC6300918

[26] Valladares-Garrido MJ Zapata-Castro LE Domínguez-Troncos H García-Vicente A León-Figueroa DA Zila-Velasque JP. Mental health disturbance after a major earthquake in northern Peru: a preliminary, cross-sectional study. Int J Environ Res Public Health. (2022) 19:8357. doi: 10.3390/ijerph19148357, PMID: 35886205 PMC9319911

[27] Rousseau C Mekki-Berrada A Moreau S. Trauma and extended separation from family among Latin American and African refugees in Montreal. Psychiatry Interpers Biol Process. (2001) 64:40–59. doi: 10.1521/psyc.64.1.40.18238, PMID: 11383441

[28] Betancourt TS Khan KT. The mental health of children affected by armed conflict: protective processes and pathways to resilience. Int Rev Psychiatry. (2008) 20:317–28. doi: 10.1080/09540260802090363, PMID: 18569183 PMC2613765

[29] Southwick SM Bonanno GA Masten AS Panter-Brick C Yehuda R. Resilience definitions, theory, and challenges: interdisciplinary perspectives. Eur J Psychotraumatol. (2014) 5:25338. doi: 10.3402/ejpt.v5.25338, PMID: 25317257 PMC4185134

[30] Vogel JM Pfefferbaum B. Family resilience after disasters and terrorism: examining the concept In: Pat-Horenczyk R Brom D Vogel J, editors. Helping children cope with trauma: individual, family and community perspectives. Routledge/Taylor & Francis Group: New York, NY, USA (2014). 81–100.

[31] Nijkrake J Gosselt JF Gutteling JM. Competing frames and tone in corporate communication versus media coverage during a crisis. Pub Rel Rev. (2015) 41:80–8. doi: 10.1016/j.pubrev.2014.10.010

[32] Ghanizadeh G Heidari M Seifi B Jafari H Pakjouei S. The effect of climate change on cardiopulmonary disease-a systematic review. J Clin Diag Res. (2017) 11:1–4. doi: 10.7860/JCDR/2017/26478.11012

